# Imaging‐Aided VT Ablation. Long‐Term Results From a Pilot Study

**DOI:** 10.1111/jce.16741

**Published:** 2025-05-26

**Authors:** Benjamin Sacristan, Hubert Cochet, Benjamin Bouyer, Romain Tixier, Josselin Duchateau, Nicolas Derval, Thomas Pambrun, Marine Arnaud, Jan Charton, Geoffroy Ditac, Allan Plant, John Fitzgerald, Soumaya Sdiri‐Cheniti, Laurens Verhaege, Michel Montaudon, Mélèze Hocini, Michel Haissaguerre, Maxime Sermesant, Pierre Jais, Frederic Sacher

**Affiliations:** ^1^ Department of Cardiac Electrophysiology Hôpital Cardiologique du Haut‐Lévêque, CHU de Bordeaux Pessac France; ^2^ IHU LIRYC, Université de Bordeaux‐Inserm Pessac France; ^3^ Department of Cardiovascular Imaging Hôpital Cardiologique du Haut‐Lévêque, CHU de Bordeaux Pessac France; ^4^ Inria, Asclepios team Sophia Antipolis France

**Keywords:** catheter ablation, CT‐Scan, imaging, InHeart software, ventricular tachycardia

## Abstract

**Background:**

Ventricular tachycardia (VT) ablation has become a cornerstone of patient care, especially for post‐MI VT. Several strategies have proven effective for achieving rhythm control in this population, but the workflow is highly variable and depends on the physician's experience.

**Aim:**

This study describes the initial systematic experience of VT ablation targeting wall thickness heterogeneity on a cardiac computed tomography (CT) scanner used as a surrogate for mapped VT isthmii.

**Methods:**

Consecutive patients with post‐MI VT, a CT scan, and a first VT ablation were included from January 2017 to May 2022. Targets were identified based on wall thickness heterogeneity. After image integration, ablation with > 10 grams, 40–50 W was performed with the aim of blocking the CT channels/render them non‐capturable. Only then was inducibility tested. Inducible VT, if any, were conventionally mapped and ablated with the aim of reaching non‐inducibility.

**Results:**

Thirty‐nine patients (97.4% male, age: mean LVEF 35 ± 10%) were included. The mean number of identified CT Channels was 3.6 ± 1.8/patient. Non‐inducibility was achieved in 19 (48.7%) of patients after initial imaging‐guided ablation, while at least one VT could be induced in 19 (48.7%). Among these patients, 4 had VT related to unblocked or reconnected CT‐determined VT channels, and 15 from other areas (border zone), typically with faster cycle length. After further mapping and ablation, 3 (7.7%) patients remained inducible. Mean radiofrequency time was 35 ± 19 min for CT Channels ablation, with an additional 11 ± 8 min for supplementary ablation (global mean RF time 35 ± 19 min). With a mean follow‐up of 47.8 ± 24.3 months, 61.9% (95% CI: 44.0%–75.5%) remained VT free.

**Conclusion:**

CT‐guided ablation represents a feasible and safe strategy for VT ablation in patients with an ischemic cardiomyopathy.

## 1 Introduction

The number of patients undergoing ventricular tachycardia (VT) ablation is growing, backed by randomized trials, leading to a class I recommendation in ischemic drug‐refractory VT [1]. Substrate ablation has demonstrated its superiority to clinical VT ablation alone [2]. However, there is no standardization of substrate ablation. It mainly relies on the electrophysiologist's experience. Substrate ablation based on cardiac MRI or CT scan [3, 4] has been described. It could be a more reproducible and exportable strategy. This study described our systematic initial experience using a workflow based on image‐guided VT ablation and its results.

Once limited to activation mapping, pacing maneuvers, and/or substrate analysis [5, 6, 7], imaging‐guided ablation procedures are now appearing as an effective way to streamline the ablation strategy and procedure workflow in ischemic and non‐ischemic cardiomyopathy. It allows precise identification of anatomy, substrate, and collateral structures for more effective and safer procedures [8, 9, 10, 11]. Furthermore, software has been developed to produce advanced 3D modeling from CT scan or MRI to identify potential arrhythmia isthmuses. These data can be implemented in electroanatomic mapping (EAM) systems to guide ablation [12, 13, 14].

Several studies indicate promising outcomes from using this type of tool as part of the ablation strategy in VT [3, 4]. The objective of this study is to present the results of our cohort in the setting of ischemic cardiomyopathy and explore the characteristics of residual VTs after an initial ablation strategy based on CT data.

## 2 Methods

### 2.1 Study Design

This study served as a pilot study to test the following image‐guided VT ablation strategy.

**Figure 1:**
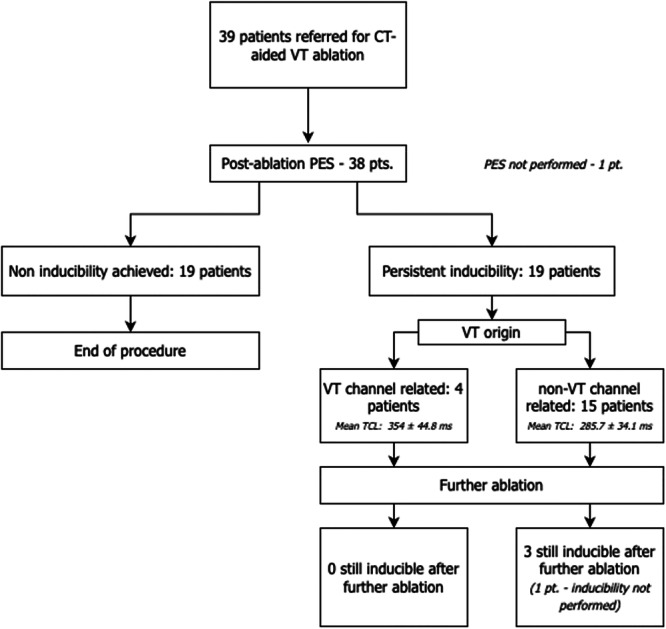
Flow chart.

**Figure 2:**
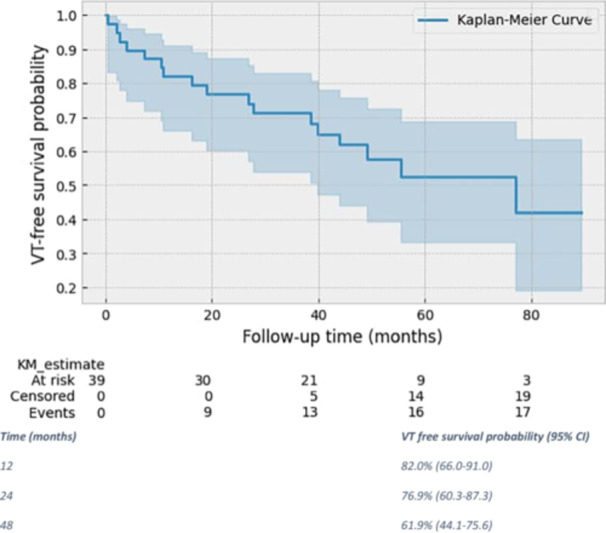
Kaplan–Meier curve of survival free of VT in the patient cohort (*n* = 39).

We included all patients referred for de novo VT ablation in the setting of ischemic cardiomyopathy who had a cardiac CT scan (Figures 1 and 2).

**Figure 3:**
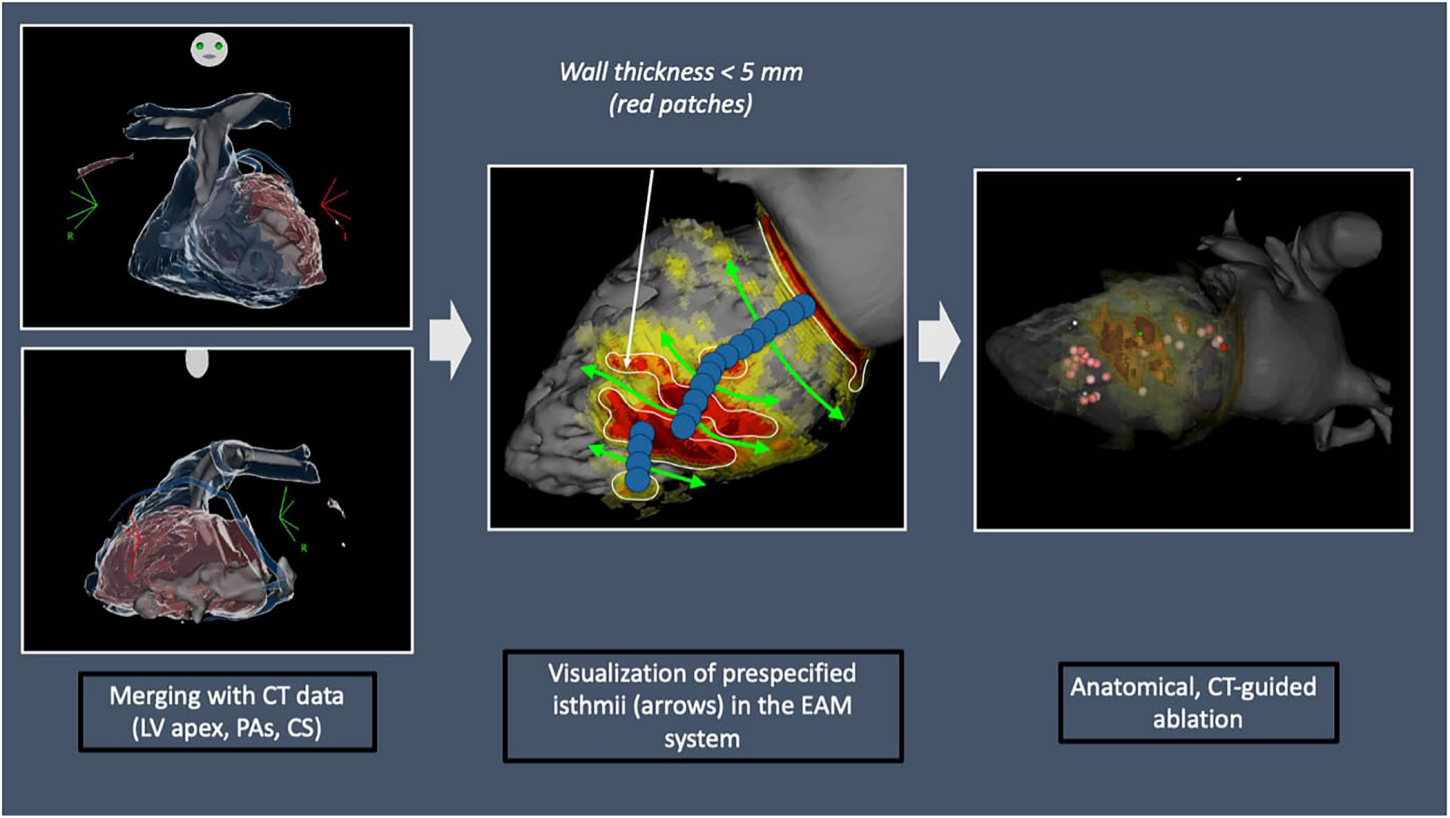
Central Figure—Description of the CT‐guided ablation workflow (ischemic VT, inferior scar). Every patient underwent a cardiac CT with segmentation using the inHeart software. CT channels were defined a priori based on wall thickness heterogeneity (wall thickness < 5 mm illustrated in red) illustrated as green arrows on the central image. The procedure starts with a quick merging step, using the CS, PA, and LV volumes. The ablation target is then defined according to the prespecified isthmii in the EAM system (Panel 2), and anatomical ablation based on these isthmii is performed (Panel 3). Inducibility is tested after this step, and further ablation is performed if still inducible.

Cardiac CT scan is systematically performed before scar‐related VT ablation in the absence of renal failure or true contrast agent allergy in our center, to rule out intracardiac thrombus [15]. The CT scan was then processed as previously reported via a dedicated software (InHeart) [16]. Briefly, cardiac anatomy was segmented, and ventricular myocardial thickness was mapped to define the MI scar area (< 5 mm). In this scar, areas from previous MI, areas of relatively preserved wall thickness are considered more likely to host surviving fibers (CT Channels, 2–5 mm) while the most severe thinned areas (1–2 mm) are more likely to act as a block/barrier during VT. The constructed 3D map was used to guide ablation to CT Channels (as a surrogate for VT isthmii). These CT channels were cross‐validated by two EPs (FS, PJ) and a radiologist (HC) (see Central Figure 3). Of note, targets were set at middle of the potential isthmii at the beginning of the study then switch to entrance and exit because of fast VT remaining inducible anchored on scar periphery and then to the thinnest part of the CT channels by the end of the study because of absence of block of the isthmus in some patients. After gaining venous and or arterial access, a quick map of the pulmonary artery bifurcation as well as the coronary sinus (for transeptal access) or the aortic arch (for retroaortic approach) was performed to allow precise merging with CT images. After accessing LV, the reliability of the merging was assessed with the ablation catheter (Termocool ST/SF, Biosense Webster) placed at the LV apex, inferior, anterior, septal, and lateral wall. CT channels were targeted using at least 10 g, with a power of 40–50 W with the aim of rendering the area non‐capturable. After ablation of all identified CT channels, inducibility using up to three extrastimuli going down to 200 ms from RV and LV was performed. If absence of inducible VT, the procedure was interrupted. In case of inducibility, the VT was targeted by activation mapping/pace‐mapping with further ablation. Inducibility was tested again up to non‐inducibility in the absence of significant concerns for continuing the procedure. Patients were then followed‐up as outpatients and via remote monitoring.

### 2.2 Data Extraction

Patients provided written informed consent for data collection in accordance with French national law (MR‐004) and recommendations from the Commission Nationale de l'Informatique et des Libertés (CNIL).

### 2.3 Cardiac CT Scan: Image Acquisition

Optimal hydration was performed before contrast injection, particularly in patients with GFR between 30 and 50 mL/min.

#### 2.3.1 Image Acquisition

The standard imaging protocol involved pre‐procedure cardiac CT (cCT) within 7 days of VT ablation. A dual‐source scanner was used for imaging (SIEMENS DEFINITION from January 2015 to March 2017; SIEMENS FORCE from March 2017 to December 2019). Contrast volume was adapted to patient weight (1 mL/kg) with a contrast media concentration of 400 mg/mL iodine for normal renal function and 350 mg/mL if impaired. A dual‐phase bolus was used (70% administered pure at 5–6 mL/s, immediately followed by 30% diluted 50/50 with saline at 5–6 mL/s). Arterial acquisition was performed at peak enhancement in the ascending aorta, with a volume comprising the whole heart and aortic arch. Tube voltage was 80–100 kV, and images were reconstructed at 0.6 mm slice thickness. Venous enhancement CT was acquired 60–90 s after contrast media injection, with a volume comprising only the heart and tube voltage lowered to 70–90 kV.

In case of intraventricular or intra‐atrial thrombus, the ablation procedure was postponed.

#### 2.3.2 Procedure Workflow

Procedures were performed under conscious sedation. Vascular access was obtained via the femoral vein and/or femoral artery. The left ventricle (LV) was accessed via a transseptal (BRK Needle, Agilis sheath, St. Jude Medical) and/or retrograde aortic approach. Following LV access, a 50 U/kg heparin bolus was administered intravenously, with an ACT target > 250–300 s, depending on the case. Merging using the pulmonary artery/aortic arch, the coronary sinus, and the LV apex, fast mapping was performed before ablation with a multipolar mapping catheter. (Biosense Pentaray/Decanav, Boston Scientific Orion or Abbott HD Grid). Initial induction was not mandatory but left to the operator's discretion. The next step consisted of ablation of the prespecified isthmuses. Then, inducibility was tested. If no VT was inducible, the procedure was ended (see Central Figure 3). If the patient was inducible, further mapping and ablation were performed if needed. Localization (isthmuses or border zone) and tachycardia cycle length (TCL) were noted. Inducibility was tested at the end of the procedure as well.

### 2.4 Follow‐Up

VT recurrence or death was assessed via telephonic interview and ICD remote monitoring reports.

### 2.5 Data Analysis

The primary endpoint was recurrent ventricular arrhythmia treated by the ICD or death. Follow‐up data were obtained at regular intervals until the date of the last follow‐up or event occurrence, whichever occurred first.

### 2.6 Statistical Analysis

Survival analysis was performed using the Kaplan–Meier estimator to calculate the probability of event‐free survival (i.e., freedom from arrhythmia recurrence or death). Survival probabilities were estimated for predefined time points (e.g., 1, 2, and 4 years) and reported with corresponding 95% confidence intervals. Missing data is reported for each variable if present.

All statistical analyses were conducted using Python (version 3.10.12) and relevant libraries, including pandas (version 2.2.2) for data extraction and analysis, Lifelines (version 0.29.0) for survival analysis, and Matplotlib (version 3.7.1) for graphical representation of the Kaplan–Meier survival curve. The survival function was plotted using standard graphical parameters. Censored patients were appropriately indicated in the Kaplan–Meier plots.

## 3 Results

### 3.1 Cohort Characteristics

Thirty‐nine patients (1 female, 64.0 ± 10.8 years old, LVEF 35.1 ± 10.1%) with ischemic CMP and de novo VT ablation were included. Two patients were excluded due to data loss (deletion of the studies due to a system failure). Baseline characteristics are described in Table 1. Ten patients (25.6%) presented with electrical storm at admission.

**Table 1:** Baseline characteristics.

		Missing data (*n*, %)
Age (mean ± SD)	64.0 ± 10.8	
Male gender (*n*, %)	38, 97.4%	
LVEF (%) (mean ± SD)	35.1 ± 10.1	
eGFR (mL/min) (mean ± SD)	82.3 ± 28.6	
Time to myocardial infarction (months) (mean ± SD)	200.9 ± 113.6	2, 5.1%
Substrate location		
Anterior/septal (*n*, %)	15, 38.5%	
Inferior (*n*, %)	33, 84.6%	
Lateral (*n*, %)	11, 28.2%	
Apical (*n*, %)	3, 7.7%	
Prior amiodarone (*n*, %)	17, 43.6%	
ICD before ablation (*n*, %)	33, 84.6%	
ICD after ablation (*n*, %)	4, 10.3%	
CRT (*n*, %)	3, 7.7%	
PCI (*n*, %)	28 (71.8%)	
CBAG (*n*, %)	9 (23.1%)	
Electrical storm at admission (*n*, %)	10 (25.6%)	

### 3.2 Substrate Characterization

CT scan was performed for every patient. Characteristics of substrate for each patient with available data are described in Table 1. The mean number of CT Channels identified was 3.6 ± 1.8.

### 3.3 Procedural Characteristics

Procedural characteristics are described in Table 2. No epicardial access was performed in this study. Mean procedural time was 197 ± 60 min. Mean fluoroscopy duration was 20 ± 7 min, and RF mean times were 41 ± 21 min.

**Table 2:** Procedural characteristics.

		Missing data (*n*, %)
Clinical VTCL (ms) (mean ± SD)	376.8 ± 67.1	9, 23.1%
Number of isthmuses identified via CT‐scan (mean ± SD)	3.6 ± 1.8	4, 10.3%
Type of access		
Transseptal access (*n*, %)	32, 82.1%	
Retro‐aortic access (*n*, %)	5, 12.8%	
Both transseptal and retro‐aortic access (*n*, %)	2, 5.1%	
Merging time (min) (mean ± SD)	9.5 ± 4.2	2, 5.1%
Pre‐ablation induction		
Not done (*n*, %)	17, 43.6%	
Done but not inducible (*n*, %)	12, 30.8%	
Via PVS (*n*, %)	7, 17.9%	
Mechanical (*n*, %)	3, 7.7%	

Ten patients underwent PES before ablation, three of them being inducible. A total of 13 patients had mechanical induction before ablation, and 17 underwent ablation without prior PES (Table 3).

**Table 3:** Ablation outcomes.

		Missing data (*n*, %)
Inducibility after first‐pass isthmus ablation		
Not inducible (*n*, %)	19, 48.7%	
Inducible (*n*, %)	19, 48.7%	
No inducibility test performed after isthmus ablation (*n*, %)	1, 2.6%	
VT origin after first‐pass ablation if inducible:		
Prespecified isthmuses (*n*, %)	4, 21.1%	
Other than prespecified isthmuses (*n*, %)	15, 78.9%	
VT cycle length after ablation (ms) (mean ± SD)	300.1 ± 45.4	
VT cycle length in cases originating from prespecified isthmuses (ms) (mean ± SD)	354 ± 44.8	
VT cycle length in cases originating from other areas (ms) (mean ± SD)	285.7 ± 34.1	
RF time for isthmus ablation (min) (mean ± SD)	35.2 ± 19.1	3, 7.7%
Additional RF time after isthmus ablation (min) (mean ± SD)	10.9 ± 8.0	3, 7.7%
Final inducibility (*n*, %)	3, 7.7%	
Total RF time (min) (mean ± SD)	40.0 ± 21.4	3, 7.7%
Procedural time (min) (mean ± SD)	196.5 ± 58.7	
Fluoroscopy time (min) (mean ± SD)	20.8 ± 7.1	19, 48.7%
AK dose (mGy) (mean ± SD)	108.9 ± 67.6	18, 46.2%
DAP (mGy/cm^2^) (mean ± SD)	14805.6 ± 17581.0	18, 46.2%

### 3.4 Initial Isthmii Ablation

Clinical VT cycle length (recorded in 30 patients) as identified by ECG/ICD recordings was 375.8 ± 65.8 ms. The mean RF time for isthmus ablation was 35 ± 19 min.

**Figure 4:**
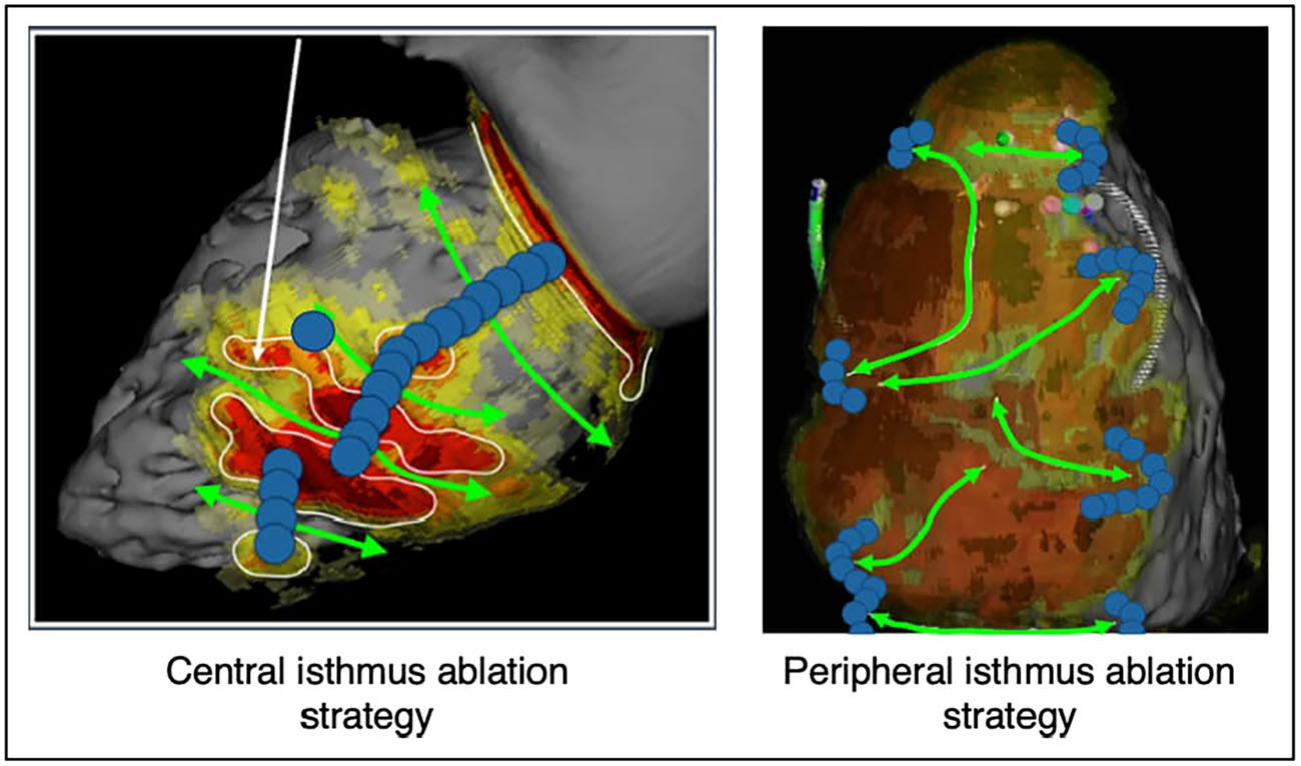
Description of the two strategies used for isthmus ablation; our main strategy consisted of central isthmus ablation. The second strategy was adopted later in the study due to fast VTs anchored in the border zone, but was finally abandoned as the isthmus block rate was inferior. Green arrows are prespecified isthmuses, blue dots show the ablation targets in each strategy.

As described earlier, we used two different strategies for isthmus ablation along the course of the study. These strategies are presented in Figure 4.

### 3.5 Inducibility After Isthmus Ablation

A total of 19 (48.7%) patients were still inducible after initial isthmus ablation. Among them, five patients had VT originating from prespecified isthmii that were either not blocked or reconnected during the procedure. VTCL in these patients was slower (354 ± 44.8 ms). For the other patients, VT originated outside of the prespecified isthmii, typically from the border zone of the scar, with faster TCL (285.7 ± 34.1 ms). Three patients were still inducible at the end of the procedure (7.7%).

### 3.6 Complications

No major complications occurred; one groin hematoma and one pseudo‐aneurysm associated with arteriovenous fistulae, both managed conservatively.

### 3.7 Follow‐Up

Figure 2 Kaplan–Meier curve details VT‐free survival for the study cohort. The mean follow‐up time was 47.8 ± 24.3 months. Six patients died of non‐arrhythmic causes, one with a history of recurrent VT treated by ICD shock. A total of 11 patients had recurrent VT. Survival probability free from arrhythmia was estimated at 82.0% at 1 year (95% CI: 66.0%–91.0%) and 61.9% (95% CI: 44.1%–75.6%) at 48 months.

## 4 Discussion

This study provides insights into the advantages and limitations of a CT‐guided approach to VT ablation in patients with ischemic cardiomyopathy. We demonstrated that this approach is safe, achieves acute non‐inducibility in about half of VT cases without further ablation and have an 82.0% absence of VT recurrence or death at 1 year and 61.9% with a mean follow‐up of 48 months, which is concordant with other data regarding catheter ablation in this population [2, 17].

### 4.1 Isthmus Definition

Since the ablation target is determined before the procedure, it is obvious that the identification of the potential isthmii condition determines the outcome of the ablation strategy. In our cohort, all ablation targets were determined by an experienced physician. This might constitute a limitation to a broader adoption of this kind of strategy in less experienced centers. However, simulation‐based models have recently shown good results in the automatic identification of VT channels related to endocardial scar [18].

CT scan was the imaging modality of choice in this study, due to its better spatial resolution compared to MRI. Photon count CT, with its increased spatial resolution, might constitute an even better imaging modality for this type of strategy, but evidence is lacking in this area.

### 4.2 Procedure Workflow

The CT‐guided procedure for VT ablation provides a standardized workflow to achieve successful ablation; merging with CT data, ablation of prespecified isthmuses, assessment of inducibility, with complementary ablation if needed.

Our method of isthmus assessment was based solely on wall thickness. It has been shown that other imaging techniques, such as perfusion imaging, could provide added value in that setting. This choice was made to provide an easier workflow for the sthmus definition. Furthermore, in ischemic cardiomyopathies, wall thickness alone is effective in defining arrhythmogenic substrate. The added value of such techniques might be more visible in the setting of non‐ischemic cardiomyopathies with intramural/epicardial substrate and without significant wall thinning.

The merging step of this approach is of particular importance to ensure a satisfying outcome. Our experience shows that a merge based on the coronary sinus/aortic root mesh as well as the LV apex is the most effective way of achieving a good merge. We found out that using atrium geometry is longer and provides more shifting of the CT model. Merging within EAM systems is an additional step, but it is typically fast, taking less than 10 min in most cases, at most 15 min. This approach is suitable for less experienced centers as it standardizes the workflow and limits procedure and RF duration.

Procedure durations align with other studies exploring this anatomical approach: Berte et al. reported a procedural time of 172 ± 48 min [4], while Englert et al. noted 158.4 ± 71.1 min in their CT‐imaging subgroups [3]. However, our study population differs significantly from previous works, focusing on first procedures on ischemic cardiomyopathies, whereas other works included redo procedures and non‐ischemic cardiomyopathies.

### 4.3 Post‐Isthmus Ablation Findings

After first‐pass ablation, we identified two types of VTs: those originating from the isthmuses and faster circuits arising from the border zone, typically unrelated to previously ablated isthmuses. These fast VTs often require additional ablation strategies. For isthmus‐related tachycardia, our ablation strategy evolved over the course of the study. The first 17 patients of the cohort were treated using a central ablation strategy, with a satisfying isthmus block rate but inducibility of rapid VTs anchored to the periphery of the scar. This led us to shift towards a more peripheral approach with the idea of providing both isthmus block and elimination of these smaller, non‐anatomically determined reentry circuits, allowing a higher non‐inducibility rate after first pass ablation. However, this strategy proved less effective in achieving complete isthmus block, likely due to the thicker myocardial wall in the border zone compared to the central scar, which reduced the likelihood of transmural lesions. This observation led us to revert to a more central isthmus ablation strategy targeting the thinnest part of the channel.

Our findings underscore the importance of [1] better defining target isthmuses before ablation to facilitate first‐pass block and increase the non‐inducibility rate, and [2] complementing this approach with additional ablation if clinically relevant VTs persist. Our data suggest that a CT‐guided approach enables rapid and effective substrate simplification by targeting potential circuits within the scar. However, it does not address VTs arising outside these isthmuses, for which an optimal strategy remains to be determined. The extent to which fast, potentially non‐sustained, and polymorphic VTs originating from the border zone should be treated remains an open question.

In our experience, these fast VT circuits are difficult to map due to their haemodynamic tolerance, but are typically anchored at the scar border zone. Given their persistence despite initial isthmus ablation, we adopted a stepwise strategy: first targeting the central isthmus and, if inducibility remained, performing border zone ablation guided by LAVA elimination and pacemapping. While this approach appears to yield the best results, further evaluation is required.

### 4.4 Study Strengths and Limitations

#### 4.4.1 Innovative and Simplified Workflow

One of the key strengths of this study is the detailed description of a standardized and reproducible workflow for CT‐guided VT ablation. This approach streamlines the procedure, reducing variability and making it accessible to centers with less experience in VT ablation. By incorporating advanced imaging tools such as the MUSIC software for pre‐procedural substrate identification, the workflow enhances precision in targeting arrhythmogenic isthmuses while optimizing safety and procedural efficiency.

### 4.5 Extended Follow‐Up Duration

With a mean follow‐up duration of 47.8 ± 24.3 months, this study provides one of the most comprehensive long‐term datasets in the field.

## 5 Limitations

### 5.1 Retrospective and Non‐Comparative Design

The retrospective nature of this study inherently introduces biases and limits the ability to draw causal inferences. Additionally, the absence of a control group precludes direct comparisons with alternative strategies, such as conventional electrophysiological approaches. Insight from the upcoming multicentric randomized inEurHeart will hopefully provide more evidence on this part.

The non‐comparative design of the study and the ablation strategy precluded any substrate mapping before ablation in our cohort, therefore, not allowing for direct comparison of isthmus identification via CT and conventional techniques. However, previous studies show that imaging provides a strong prediction of anatomical isthmuses without the need of thorough mapping. The fact that half of the patients are non‐inducible after CT‐defined isthmus ablation supports this hypothesis. This allows for simpler and shorter procedures, sparing initial mapping time and catheter manipulation in the LV. Some previous work showed that catheter manipulation for substrate mapping could suppress or alter VT inducibility [19], so our strategy might prove beneficial to address this issue.

### 5.2 Monocentric Cohort

Being a single‐center study, the findings may lack generalizability, particularly to lower‐volume centers or those with different patient populations. Although the standardized nature of the workflow helps mitigate this limitation, external validation in multicenter settings is essential to confirm its broader applicability.

### 5.3 Missing Data

The study was constrained by missing data, particularly in relation to clinical VT characterization and radioprotection data, which is an inherent limitation of retrospective analyses.

### 5.4 Heterogeneity in Ablation Strategies

The evolution of the ablation strategy during the study period, from mid‐isthmus to entrance/exit targeting and back, introduces some variability in the approach. While this reflects an adaptive learning curve, it complicates the assessment of the true efficacy of a single standardized protocol.

### 5.5 Lack of Advanced Imaging Integration and New Ablation Technologies

While CT was chosen for its superior spatial resolution and its wide availability, the absence of comparative data with other imaging modalities, such as MRI or photon‐counting CT, limits the ability to assess whether the observed outcomes represent the optimal imaging‐guided strategy. Furthermore, we have seen in the past few years an increasing interest in new technologies such as pulsed field ablation, which constitutes a promising new tool to provide better outcomes for VT ablation, and its integration in this workflow remains to be explored.

## 6 Conclusion

CT‐guided VT ablation is a feasible, safe, and effective method to treat patients with ischemic cardiomyopathy presenting for a first VT ablation.

## Conflicts of Interest

The authors declare no conflicts of interest.

## Data Availability

The data that support the findings of this study are available from the corresponding author upon reasonable request.

## References

[1] K. Zeppenfeld , J. Tfelt‐Hansen , M. De Riva , et al., “2022 ESC Guidelines for the Management of Patients With Ventricular Arrhythmias and the Prevention of Sudden Cardiac Death,” European Heart Journal 43, no. 40 (2022): 3997–4126. 36017572 10.1093/eurheartj/ehac262

[2] L. Di Biase , J. D. Burkhardt , D. Lakkireddy , et al., “Ablation of Stable VTs Versus Substrate Ablation in Ischemic Cardiomyopathy,” Journal of the American College of Cardiology 66, no. 25 (2015): 2872–2882. 26718674 10.1016/j.jacc.2015.10.026

[3] F. Englert , F. Bahlke , N. Erhard , et al., “VT Ablation Based on Ct Imaging Substrate Visualization: Results From a Large Cohort of Ischemic and Non‐Ischemic Cardiomyopathy Patients,” Clinical Research in Cardiology: Official Journal of the German Cardiac Society 113, no. 10 (2024): 1478–1484. 38112744 10.1007/s00392-023-02321-1 PMC11420303

[4] B. Berte , H. Cochet , L. Dang , et al., “Image‐Guided Ablation of Scar‐Related Ventricular Tachycardia: Towards a Shorter and More Predictable Procedure,” Journal of Interventional Cardiac Electrophysiology 59, no. 3 (2020): 535–544. 31858334 10.1007/s10840-019-00686-w

[5] A. L. Waldo and R. W. Henthorn , “Use of Transient Entrainment During Ventricular Tachycardia to Localize a Critical Area in the Reentry Circuit for Ablation,” Pacing and Clinical Electrophysiology: PACE [Internet] 12, no. 1 Pt 2 (1989): 231–244, https://pubmed.ncbi.nlm.nih.gov/2466258/. 2466258 10.1111/j.1540-8159.1989.tb02652.x

[6] F. Sacher , H. S. Lim , N. Derval , et al., “Substrate Mapping and Ablation for Ventricular Tachycardia: The LAVA Approach,” Journal of Cardiovascular Electrophysiology [Internet] 26, no. 4 (2015): 464–471, https://pubmed.ncbi.nlm.nih.gov/25328104/. 25328104 10.1111/jce.12565

[7] P. Jaïs , P. Maury , P. Khairy , et al., “Elimination of Local Abnormal Ventricular Activities,” Circulation 125, no. 18 (2012): 2184–2196. 22492578 10.1161/CIRCULATIONAHA.111.043216

[8] E. Perez‐David , Á. Arenal , J. L. Rubio‐Guivernau , et al., “Noninvasive Identification of Ventricular Tachycardia‐Related Conducting Channels Using Contrast‐Enhanced Magnetic Resonance Imaging in Patients With Chronic Myocardial Infarction,” Journal of the American College of Cardiology 57, no. 2 (2011): 184–194. 21211689 10.1016/j.jacc.2010.07.043

[9] J. A. White , N. M. Fine , L. Gula , et al., “Utility of Cardiovascular Magnetic Resonance in Identifying Substrate for Malignant Ventricular Arrhythmias,” Circulation Cardiovascular Imaging 5, no. 1 (2012): 12–20. 22038987 10.1161/CIRCIMAGING.111.966085

[10] S. Yamashita , F. Sacher , S. Mahida , et al., “Role of High‐Resolution Image Integration to Visualize Left Phrenic Nerve and Coronary Arteries During Epicardial Ventricular Tachycardia Ablation,” Circulation Arrhythmia and Electrophysiology 8, no. 2 (2015): 371–380. 25713213 10.1161/CIRCEP.114.002420

[11] L. Y. Lin , M. Y. Su , J. J. Chen , et al., “Conductive Channels Identified With Contrast‐Enhanced MR Imaging Predict Ventricular Tachycardia in Systolic Heart Failure,” JACC Cardiovascular Imaging 6, no. 11 (2013): 1152–1159. 24229767 10.1016/j.jcmg.2013.05.017

[12] S. Yamashita , F. Sacher , S. Mahida , et al., “Image Integration to Guide Catheter Ablation in Scar‐Related Ventricular Tachycardia,” Journal of Cardiovascular Electrophysiology 27, no. 6 (2016): 699–708. 26918883 10.1111/jce.12963

[13] N. Cedilnik , J. Duchateau , R. Dubois , P. Jaïs , H. Cochet , and M. Sermesant , “VT Scan: Towards an Efficient Pipeline From Computed Tomography Images to Ventricular Tachycardia Ablation.” in Functional Imaging and Modelling of the Heart, eds. M. Pop and G. A. Wright (Springer International Publishing, 2017), 271–279).

[14] H. Cochet , Y. Komatsu , F. Sacher , et al., “Integration of Merged Delayed‐Enhanced Magnetic Resonance Imaging and Multidetector Computed Tomography for the Guidance of Ventricular Tachycardia Ablation: A Pilot Study,” Journal of Cardiovascular Electrophysiology 24, no. 4 (2013): 419–426. 23252727 10.1111/jce.12052

[15] T. Bonnin , P. Roumegou , S. Sridi , et al., “Prevalence and Risk Factors of Cardiac Thrombus Prior to Ventricular Tachycardia Catheter Ablation in Structural Heart Disease,” EP Europace 25, no. 2 (2023): 487–495. 36355748 10.1093/europace/euac156 PMC10103557

[16] M. Merle , F. Collot , J. Castelneau , et al., “MUSIC: Cardiac Imaging, Modelling and Visualisation Software for Diagnosis and Therapy,” Applied Sciences 12, no. 12 (2022): 6145.

[17] J. L. Sapp , A. S. L. Tang , R. Parkash , W. G. Stevenson , J. S. Healey , and G. Wells , “A Randomized Clinical Trial of Catheter Ablation and Antiarrhythmic Drug Therapy for Suppression of Ventricular Tachycardia in Ischemic Cardiomyopathy: The VANISH2 Trial,” American Heart Journal 274 (2024): 1–10. 38649085 10.1016/j.ahj.2024.04.009

[18] N. Cedilnik , M. Pop , J. Duchateau , et al., “Efficient Patient‐Specific Simulations of Ventricular Tachycardia Based on Computed Tomography‐Defined Wall Thickness Heterogeneity,” JACC: Clinical Electrophysiology 9, no. 12 (2023): 2507–2519. 37804259 10.1016/j.jacep.2023.08.008

[19] A. A. Aboud , G. Davogustto , O. Adeola , et al., “Substrate Mapping Alters Ventricular Tachycardia Inducibility,” Circulation Arrhythmia and Electrophysiology 16, no. 2 (2023): e010889. 36602818 10.1161/CIRCEP.122.010889

